# Digital information manipulation, AI readiness, and security outcomes in Africa: evidence from Africa’s most populous countries

**DOI:** 10.3389/fsoc.2026.1964645

**Published:** 2026-10-08

**Authors:** Tersoo Solomon Shaminja, Merlyn Barkhuizen

**Affiliations:** Department of Criminology and Security Science, University of South Africa, Pretoria, South Africa

**Keywords:** information security, digital information manipulation, AI readiness, disinformation, Africa

## Abstract

**Introduction:**

The advent of generative AI has reduced costs, scaled up operations, and made it more difficult to identify disinformation and manipulated content, leading to new governance and security challenges in Africa. This paper analyses the nexus among the manipulation of digital information, governments’ readiness to adopt AI, digital connectivity, and security outcomes in Africa’s five most populous countries, namely Nigeria, South Africa, Egypt, Ethiopia, and the Democratic Republic of Congo, for the period from 2023 to 2026. The research attempts to fill a gap in the existing literature by addressing the lack of empirical research on the relationship between digital information manipulation and government AI readiness, and on its consequences for security issues in Africa.

**Methods:**

The paper draws on the theoretical frameworks of Social Learning Theory and Routine Activity Theory and uses seven credible datasets, including ACLED, UCDP/PRIO, V-Dem, GSMA, ITU, the Oxford Insights Government AI Readiness Index, and Afrobarometer, to construct a country-year panel. Correlation analyses were conducted to test three hypotheses regarding the relationship among digital manipulation, digital connectivity, institutional readiness for AI adoption, and conflict fatalities. It utilises a verified multisource panel dataset spanning five countries and operationalises AI readiness as an institutional capability distinct from digital exposure.

**Results:**

Digital manipulation was found to have a weak positive correlation with fatalities (*r* = 0.23), a moderate negative correlation with digital connectivity (*r* = −0.56), and a weak negative correlation with institutional readiness to adopt AI (*r* = −0.42). These findings show that correlations between digital exposure and security consequences may be distorted by underlying conflict contexts.

**Discussion:**

It can therefore be deduced that although digital manipulation is becoming an emerging security challenge, conflict and other structures are more significant predictors of security outcomes. This study offers an initial cross-national empirical assessment of AI-related security vulnerabilities in Africa and calls for stronger AI governance and longitudinal, event-level research.

## 1 Background

Security in contemporary Africa is shaped as much by what happens on screens as by what happens on the ground. Generative artificial intelligence (AI) has revolutionised the way information is generated, appearing at a pace and scale unimaginable 10 years ago, creating convincing text, images, audio and video. While these capabilities present significant economic and social opportunities, they have also enabled misinformation, disinformation, deepfakes and hate-based narratives that can affect public opinion and challenge social relationships. Therefore, in this study, digital information manipulation is considered the first independent variable, defined as the deliberate dissemination of false or misleading information by governments, political actors, or other organised groups via digital platforms (Hunter et al., 2024). The second relates to government AI readiness, which refers to the institutional, infrastructural and policy capabilities of the government to govern, regulate and responsibly deploy AI. These variables are not the same but are related. Manipulation of Digital information is a threat to security, and AI readiness is a country’s ability to anticipate and manage it.

The safety concerns of AI-powered manipulation are already apparent in the world’s political systems. AI technologies are increasingly being incorporated into information warfare and influence operations, thereby enhancing nations’ ability to manipulate information to achieve strategic goals in the digital domain. The evidence from America, China, and Russia indicates the use of AI in information-war strategies consistent with national security, highlighting the importance of AI-enabled information manipulation from a security perspective (Hunter et al., 2024). The Progressive Slovakia party’s leader was impersonated in an audio deepfake that accused him of voting-buying and rigging the elections, days before Slovakia’s 2023 parliamentary election. The recording was later discredited, but it did erode confidence in the electoral process (Wirtschafter, 2024). In another year, AI-driven influence campaigns were responsible for overturning the results of Romania’s presidential election, marking the largest known instance of AI-powered electoral manipulation at the time (McKay and Trauthig, 2025). This is evidence that fake media has evolved from being merely a potential threat of technology to being a tool for disrupting democracy.

These technologies have their own social and political conditions that affect their adoption in Africa, such as ethnic diversity, religious plurality, weak democracy, and security concerns. Propaganda technology based on AI is currently being used in Nigeria, Burkina Faso and Gabon to affect the politics during key moments of democratic interaction (Okolo, 2024). President of Nigeria, Bola Tinubu, appeared in an audio clip that was believed to be recorded using an AI-generated voice of him, but in reality, it was the voice of an activist named Martins Vincent Otse (“VeryDarkMan”), which caused the calls for prosecution and public protests both online and offline (BBC News Pidgin, 2026). This incident is similar to the one which happened just before the 2023 presidential elections in Nigeria, when a viral video of the candidate Atiku Abubakar and his Vice-Presidential candidate, Ifeanyi Okowa stated that there was a plot to manipulate the results of the election, which worried many Nigerians less than a day before the voting began (Vanguard, 2023).

Similar trends have been seen in other parts of Africa. In South Africa, a deepfake video of former U.S. President Donald Trump endorsing the Umkhonto we Sizwe party during the 2024 election was among the most widely shared AI-generated videos circulating during the elections (Deutsche Welle, 2025). In Kenya, anti-government protesters were shown in images to be holding Russian flags, believed to be a smear campaign by the government against the protests (Chenrose and Rizzuto, 2025). In Burkina Faso, a similar trend was observed, with fake videos of the coup leader, Captain Ibrahim Traoré, shaking hands with world leaders and even pro-Russian messages sweeping across Nigeria, Ghana, and Kenya. The narratives glorified the junta and spread misinformation beyond Burkina Faso (BBC News Pidgin, 2025).

The question of government readiness is posed by this growing threat, as African nations are currently grappling with protecting their information environments from the manipulative power of AI while building the policies, infrastructure, and human resources needed to govern AI more broadly. This capacity is currently assessed across 195 countries using indicators derived from the Oxford Insights Government AI Readiness Index (7th edition), which categorises countries’ readiness levels in policy vision, technical infrastructure, and the adoption of AI in public services (Oxford Insights, 2025). But it is not known whether this preparedness leads to tangible advances in security outcomes. The key empirical question that motivates the present study is the establishment of this relationship.

### 1.1 Research gaps

However, the current knowledge of this relationship in Africa is limited by three important gaps. First, much of the literature on risks associated with AI treats digital manipulation as part of campaigns of electoral interference and/or disinformation, without focusing specifically on it as a measurable security phenomenon (Fournier-Tombs, 2023). Secondly, the current scholarship is largely shaped through media, communication science, and political science lenses, with the criminological and security aspects of AI-manipulated media comparatively under-researched (Al-Raggad, 2026). Third, and most crucially, there is no systematic study that logically ties the manipulation of digital information and governments’ readiness for AI as two measures, together predicting documented security outcomes across several African countries, based on verified secondary data. Existing research on Africa is largely confined to case studies of election periods in a single country (Shaminja et al., 2026). The gaps and challenges addressed in this study include the use of harmonised panel data from various sources, such as ACLED, V-Dem and the Government AI Readiness Index, which enables comparative analysis of these relationships.

### 1.2 Statement of the problem

Africa is a fast-growing digital market. It is also a region where structural factors such as weak regulatory capacity, contested elections, active conflicts, and even deep ethnic and religious divisions can make digital information manipulation particularly consequential. Governments also have a wide range of capabilities to govern and address new risks arising from AI. Others have put in place relatively mature AI governance frameworks, while others have limited institutional capacity.

Despite the different contexts, there is insufficient empirical evidence to establish a link between a nation’s performance in digital information manipulation and security outcomes, or between a nation’s AI readiness and security outcomes. There is a lot of information from qualitative assessments and case studies on the elections. Therefore, a supposed link between digital manipulation, government readiness, and security remains untested. It hinders both theoretical development and evidence-based policymaking. The current research directly addresses this issue by examining these links using available secondary data from the five most populous African nations.

### 1.3 Research objectives

This paper attempts to achieve three aims:

1. Analyse the link between digital manipulation and security impacts in the five most populous African states.
2. Assess how digital exposure, measured through connectivity and platform access, relates to the severity of security outcomes across the study countries.
3. Evaluate the relationship between government AI readiness and the severity of documented security outcomes and examine its implications for security governance.

### 1.4 Research hypotheses

These two constructs, digital information manipulation and digital exposure (connectivity), are treated as independent variables in the study; government AI readiness is an institutional capacity variable, while the security outcomes measured include conflict-related fatalities and events (dependent variables). Three hypotheses that will serve the research objectives include:

- H1: Digital information manipulation intensity is positively associated with the severity of recorded security outcomes across the study countries.
- H2: Digital exposure is positively associated with the severity of documented security outcomes across the study countries.
- H3: Government AI readiness is negatively associated with the severity of documented security outcomes across the study countries.

### 1.5 Contribution of this study

This study has three specific contributions to the literature covered in Section 1.2: studying the effects of digital information manipulation, digital exposure, and government AI readiness on security outcomes using a verified, multi-source, cross-national panel rather than a single-country case study. Second, the study empirically demonstrates that government AI readiness is only loosely related to digital exposure (Section 6.4) and treats it as a separate institutional-capacity factor, a distinction not made by single-variable “digital development” indices. Third, the analysis highlights a methodological caveat with direct policy implications. The correlation between exposure and fatalities was negative at the bivariate level (Section 6.2), but this does not indicate a protective effect of connectivity; rather, it reflects the confounding effect of conflict status on the correlation (Section 6.3).

## 2 Literature review

The literature is structured around the study’s three research objectives, and each objective is supported by its own specific yet related literature. The review explores the outcomes of digital information manipulation and security, the potential of digital exposure as an enabling or mitigating condition, and the institutional capacity of governments to prepare for AI readiness as a basis for security governance. The theoretical framework then helps to synthesise these strands into a coherent analytical perspective.

### 2.1 Effects of digital information manipulation and security outcomes

The advent of Artificial Intelligence (AI) has not only increased the amount of bad content online but also created a powerful tool for detecting it, making it a truly double-edged sword. As AI-generated hate speech becomes increasingly difficult to spot among other texts, increasingly complex detection technologies are required to detect it successfully (Chaturvedi et al., 2026). However, the problem is not confined to texts. Hateful images generated by AI may effectively “hide hate in plain sight,” thereby bypassing filters designed to detect other forms of hate speech (Qu et al., 2025).

In criminology, such a phenomenon has been described as the “industrialisation of deception”, in which deepfakes, voice cloning, synthetic identities, and automated phishing, once used only by sophisticated perpetrators, are increasingly becoming part of a developing industry of deception (Bailo et al., 2026). In other words, generative AI makes it cheaper and easier to create misleading information, thus allowing new participants in deceptive activities to enter the market (Bashardoust et al., 2024). Therefore, the power of deception and the ability to produce misleading content have been scaled to a new level.

However, one significant empirical gap in our knowledge of digital information manipulation remains: Does greater exposure to manipulation lead to deterioration in security outcomes? While there is ample empirical research documenting the technological capacity to engage in deception, most of it focuses on individual incidents, election-period manipulations, and platform-specific cases.

### 2.2 Digital exposure and security outcomes

The security impacts of AI system manipulation will depend in part on the extent and context of digital exposure. AI systems can take advantage of features inherent in human decision-making, such as bounded rationality and heuristics and biases, including confirmation bias, to influence judgment (Matz et al., 2024). The use of these characteristics to influence judgments is especially relevant in AI systems used in large-scale settings, compared with individual cases.

From an international perspective, information manipulation through AI has been framed as part of a broader security dilemma involving instability in deterrence, potential escalation, and structural vulnerability (Johnson, 2022). Research on information warfare has further posited that digital infrastructures developed for optimal connectivity and efficiency will create vulnerabilities that adversaries can exploit precisely using AI technology (Hunter et al., 2024). The risks associated with these factors, however, will vary with the levels of digital governance, cybersecurity, access to protection, and AI technology. As such, these risks might make countries in the Global South, including Africa, vulnerable to cybercrime and AI manipulation (Kshetri, 2019; Okolo, 2024). Exposure to digital platforms, however, should not necessarily have negative impacts. It might enhance connectivity to fact-checking, information, institutions, education and economic opportunities. Therefore, while there is potential for manipulation, there is also potential for resistance.

### 2.3 Government AI readiness and security governance

The capacity of governments to govern AI is increasingly recognised as a distinct dimension of public-sector capability, separate from connectivity and infrastructure development. Within the realm of non-consensual deepfakes and disinformation campaigns, current international efforts to counteract these phenomena are hampered by the lack of binding global guidance, weak accountability, and limited remedy options available to victims of disinformation and deepfakes (Ahmad, 2026). As regards vulnerabilities in enforcement and ambiguities in regulation identified through legislative approaches across different states, these gaps can be exploited by malicious individuals or groups. Without a universal regulatory framework applicable in all cases, institutional capacity will likely be a stronger predictor of a country’s ability to address AI-related challenges (Xu, 2026).

The issue of AI governance also extends beyond the public social media sphere. With advances in Artificial Intelligence, increased hate speech and manipulations are emerging, which can penetrate the communication channels of organisations and institutions, disrupting conventional communication (Żywiołek, 2025). From the perspective of platforms, one main structural challenge remains. The regulatory mechanisms and rules that work for certain kinds of content and certain platform architectures fail to adapt to the constantly emerging AI-generated content (He and Fang, 2024).

Therefore, according to the literature, the efficiency of AI governance depends not only on the presence of regulation itself but also on the institution’s capacity to anticipate and respond to potential risks associated with it. Such an approach provides justification for using government readiness for AI as a proxy variable for assessing institutional capacity.

## 3 Theoretical framework

### 3.1 Conceptualising digital information manipulation, AI readiness and security

The present study views the modern digital space as a potential source of criminality, in which technological, social, and organisational aspects combine to create motivations and opportunities for engaging in problematic information practices. The concept draws initially on Merton’s anomie and strain theory, which explains deviant behaviour in terms of the tension between culturally valued goals and unequal access to legitimate means of achieving them (Merton, 1938). Applied to digital environments, this perspective suggests that information systems may become criminogenic when manipulation, deception, or disinformation is perceived as an effective or acceptable means of achieving desired political or social objectives (Apel and Paternoster, 2009). Sutherland’s differential association theory informs this idea, which holds that definitions of criminal behaviour, whether positive or negative, are learned from others (Sutherland, 1947). Within the online world, these definitions circulate fast due to networked communication and frequent exposure to manipulation.

Government AI readiness is conceptualised separately as an institutional-capacity variable, consistent with the Oxford Insights framework. It refers to the combined policy, infrastructural, technical, and governance capacity through which states anticipate, regulate, and respond to AI-related risks (Oxford Insights, 2025). Security is defined broadly as a condition in which individuals, communities, and states are protected from threats to social cohesion, public order, political stability, and physical safety (Jore, 2019; Taylor, 2024; Olteanu, 2024; Reinsberg et al., 2024). Empirically, this study operationalises security through documented conflict events and fatalities.

### 3.2 Social learning theory

Social Learning Theory (SLT) holds that behaviour is acquired through observation, imitation, and reinforcement within a social environment (Bandura, 1977). Behaviour is therefore not simply an individual choice; it can be learned and reinforced through exposure to models and the consequences associated with their behaviour. The contemporary formulation of SLT is principally associated with Albert Bandura, whose work demonstrated how individuals can learn behaviour through observing others and through subsequent reinforcement.

Three specific mechanisms relevant to this study are: observational learning, in which behavioural patterns are learned through observing others; imitation, which involves replicating observed patterns; and reinforcement, which includes rewards, approval from others, and repetition to enhance learned behaviour (Bandura, 1977). Modern theory applies these mechanisms to technological media, where learning occurs through symbolic models provided by algorithms in social media and computer-mediated communication (Holt et al., 2010). Repetition and interaction with these platforms can strengthen behavioural patterns, while algorithmic amplification can make certain types of content more frequent and more widely available (Holt et al., 2010).

Although Social Learning Theory is useful for explaining how exposure, observation, imitation, and reinforcement can shape behaviour, its primary explanatory emphasis is on the processes through which behaviour is learned rather than on the structural and situational conditions that create opportunities for harmful behaviour. Research applying social learning approaches to cyber-deviance likewise demonstrates their value in explaining behavioural processes and highlights the importance of broader social and structural conditions (Bandura, 1977; Villamil and King, 2024). In the context of digitally mediated manipulation, therefore, SLT is better complemented by an opportunity-based perspective that can account for target exposure, environmental conditions, and also the presence or absence of capable guardianship. Routine Activity Theory provides this complementary perspective (Cohen and Felson, 1979).

SLT provides the theoretical basis for understanding digital information manipulation as a potential mechanism through which behavioural norms and harmful narratives can be learned and reinforced. Digital platforms can serve as symbolic environments where disinformation, emotionally charged political narratives, and other manipulative content are repeatedly encountered. Where such content receives attention, engagement, or social reinforcement, repeated exposure may contribute to its normalisation and potential reproduction. Accordingly, SLT supports the expectation that greater digital information manipulation will be associated with more severe security outcomes (H1) and that greater digital exposure will increase the population that can be reached by manipulative content (H2).

### 3.3 Routine activity theory

Routine Activity Theory (RAT) explains crime as the outcome of the convergence of three elements: a motivated offender, a suitable target, and the absence of capable guardianship (Cohen and Felson, 1979). Rather than focusing exclusively on offender motivation, RAT emphasises the situational conditions that create opportunities for harmful behaviour. RAT was formulated by Cohen and Felson (1979); their framework focused on routine activities and environmental settings that create opportunities for criminal activity. The basic idea is that crimes occur when motivated offenders find targets lacking competent guardianship (Cohen and Felson, 1979). Thus, any changes in the environment and routines can change the opportunities for the commission of crime.

RAT was developed to explain conventional crime and, although it has been adapted to cybercrime, its explanatory components do not operate uniformly in digital environments (Holt et al., 2020). Its focus on situational opportunity and guardianship also provides limited insight into how individuals learn, reproduce, and reinforce manipulative behaviours and narratives. Integrating RAT with SLT, therefore, provides a stronger framework by combining opportunity and guardianship with learning and reinforcement processes.

RAT provides the opportunity-based explanation for how digital manipulation may translate into security risks. A motivated offender is an individual or organisation that has both the means and the capability to use AI-generated manipulation. Suitable targets would be individuals who are highly exposed to digital media and have difficulties detecting manipulated content. The guardianship would involve institutional measures to detect, control, and handle threats related to AI technology. Government AI readiness can thus be seen as one aspect of guardianship. In the absence of adequate government readiness, institutional capacity to detect and constrain manipulation may be lacking, thereby increasing the likelihood that digital manipulation will lead to harmful consequences. This is because of evidence in the cybersecurity literature on the gap between technological developments and institutional capacity, which creates opportunities for cybercrime and other digitally assisted crimes (Holt et al., 2020; Maimon et al., 2023). Thus, RAT provides the theoretical basis for anticipating a relationship between higher government AI readiness and lower security outcomes (H3).

### 3.4 Integration of the theoretical framework

SLT and RAT provide explanations for different, but complementary aspects of the relationship under investigation in this study. SLT explains the learning process, while RAT explains the opportunity and guardianship aspects of the process.

The combination of the two theories creates a consistent analytical framework. Digital manipulation creates harmful content; digital exposure broadens its reach; and government AI readiness represents an institutional capability for guardianship. In cases of frequent exposure to manipulative content, SLT indicates a higher likelihood of internalising or reproducing it. In cases where there is extensive digital exposure but limited guardianship capability, RAT shows a higher risk of manipulation causing harm. On the contrary, high institutional readiness should limit the transformation of digital manipulation into physical insecurity.

Thus, the resulting research framework yields the following hypotheses: H1, a positive relationship between digital information manipulation and security outcomes; H2, a positive relationship between digital exposure and security outcomes; and H3, a negative relationship between government AI readiness and security outcomes. Subsequently, theoretical expectations are being tested empirically by using verified secondary data.

## 4 Methodology

### 4.1 Research design

This study is a quantitative (country-year) comparative study using only validated secondary data. The analysis will be exploratory and descriptive-correlational, examining correlations among the manipulation of digital information, digital exposure, government AI readiness, and security outcomes. The design cannot be used for causal inference due to the limited number of countries and the short time span. The analysis is thus directed toward the patterns of association and their strength for different measures and specifications.

### 4.2 Study setting and case selection

A purposive sampling approach has been used to investigate Nigeria, South Africa, Egypt, Ethiopia, and the Democratic Republic of the Congo (DRC), the five largest African nations by population. Population size is an objective and replicable case-selection criterion, thereby ensuring the inclusion of states with large populations and varying levels of digital connectivity, information environment, institutional capabilities, AI readiness, and security situation. Therefore, the selected states will provide the basis for an intended cross-national comparison, and not a representative sample of all African nations. It is also worth mentioning that the five selected countries cannot be considered as a representative sample of Africa or a selected sample with high/low levels of AI readiness. The period under investigation goes from 2023 to 2026, depending on the time coverage of particular data sets. ACLED and UCDP/PRIO (Uppsala University, Department of Peace and Conflict Research, 2026) go to 2026; V-Dem Institute, University of Gothenburg, (2026) and (GSMA, 2025) cover the period up to 2025; while the Oxford Insights Government AI Readiness Index gives values for 2025. Afrobarometer Round 9 includes national-level data collected between March 2022 and June 2023. Thus, the analytic sample varies by analysis due to differences in variable coverage.

### 4.3 Data sources and data preparation

Security outcomes were drawn from seven secondary data sets: the Armed Conflict Location and Event Data Project, (2026), Armed Conflict Dataset (ACD) from the University of Colorado, Denver (UCDP/PRIO) for robustness checks on conflict outcomes; the V-Dem Digital Society Project for digital information manipulation; the Mobile Connectivity Index (MCI), and the International Telecommunication Union (2026) ICT indicators for digital exposure; and the Afrobarometer Round 9 for supplementary robustness measures of public digital and media use (Afrobarometer Network, 2024). Datasets were harmonised by country and year, and checked for consistency, temporal coverage, missing observations, and completeness before analysis. All variables were matched at the country-year level, and all analyses were limited to country-years with available measures.

### 4.4 Analytical unit and sampling

The study did not involve respondent-level sampling since it is an aggregate secondary study. The five countries were selected based on the criterion of population size and they also included variation in the principal explanatory and outcome conditions. The country-year observation is the unit of analysis, with a maximum of 20 observations in the five countries for 2023–2026. The effective sample varies by analysis, as each analysis has a different temporal coverage of the source datasets.

### 4.5 Variables and operationalisation

Table 1 summarises the study variables, their operationalisation, and data sources.

**Table 1:** Variables and operationalisation.

Variable	Operationalisation	Source
Security outcomes (DV)	Annual sum of documented fatalities per country (ACLED)	ACLED
Armed conflict (robustness)	Count of active conflict-dyads; maximum intensity level reached per country-year	UCDP/PRIO ACD
Digital information manipulation (IV)	Mean of reverse-coded government domestic disinformation, foreign government disinformation, and party hate-speech scores	V-Dem Digital Society Project
Digital exposure (IV)	Mean of the Mobile Connectivity Index score and the household internet-access percentage	GSMA MCI; ITU
Government AI readiness (moderator)	Total Government AI Readiness Index score and 6 pillar sub-scores	Oxford Insights GARI
Public perceptions (optional robustness)	% reporting frequent social-media news use and frequent internet use	Afrobarometer R9

### 4.6 Construction of composite measures

Two composite indices were constructed using equal weighting. The Digital Information Manipulation Index (DIM) combines three V-Dem indicators measuring domestic government disinformation, foreign government disinformation targeting the country, and political-party hate speech:

$${\mathrm{DIM}}_{\mathrm{it}}=\frac{v2\text{smgovdo}m_{\mathrm{it}}+v2\text{smfordo}m_{\mathrm{it}}+v2\text{smpolhat}e_{\mathrm{it}}}{3}$$ (1)

where i denotes country and t year. Component indicators were reverse-coded where necessary so that higher values consistently indicate greater digital information manipulation.

The Digital Exposure Index (DEI) combines the GSMA Mobile Connectivity Index (MCI) and ITU household Internet access:

$${\mathrm{DEI}}_{\mathrm{it}}=\frac{\mathrm{MC}I_{\mathrm{it}}+\text{InternetAcces}s_{\mathrm{it}}}{2}$$ (2)

Both variables are scaled from 0 to 100, with a higher value indicating greater digital connectivity and access. Equal weighting of both measures creates a transparent index that reflects the enabling environment for connectivity and the actual household Internet access. The component measures were used for robustness analysis.

The security outcomes are measured by annual deaths reported by the ACLED database. The log transformation of fatalities was considered in the sensitivity analysis to reduce the effect of extreme values at the country level, particularly in Nigeria and the Democratic Republic of Congo. The overall Oxford Insights score measures AI readiness, while its six pillars are used for descriptive purposes only and serve as additional measures. AI readiness is considered an institutional capacity indicator and not a moderator of effects.

### 4.7 Data analysis

Country-year observations from the available datasets were harmonised and merged into a country-level analytical panel. Descriptive statistics were first calculated for each variable by country and year to assess distributions and cross-country variation. The principal analysis used bivariate Pearson correlation coefficients to examine associations among the Digital Information Manipulation (DIM) Index, Digital Exposure Index, Government AI Readiness Index, and ACLED fatalities. Because the source datasets have different temporal coverage, pairwise-complete observations were used for associations involving multi-year variables. The AI-readiness analysis is restricted to 2025 because the 2025 Government AI Readiness Index provides one observation per country. Correlations are interpreted as descriptive associations in direction and magnitude, not as evidence of causality or statistical significance, given the small and varying sample sizes.

Country-year observations from the available datasets were harmonised and merged into a country-year analytical panel. Descriptive statistics were first calculated for each variable by country and year to assess distributions and cross-country variation. The principal analysis used bivariate Pearson correlation coefficients to examine associations among the Digital Information Manipulation (DIM) Index, Digital Exposure Index, Government AI Readiness Index, and ACLED fatalities. Because the source datasets have different temporal coverage, pairwise-complete observations were used for associations involving multi-year variables. The AI-readiness analysis is restricted to 2025 because the 2025 Government AI Readiness Index provides one observation per country. Correlations are interpreted as descriptive associations in direction and magnitude, not as evidence of causality or statistical significance, given the small and varying sample sizes.

#### 4.7.1 Data validation and robustness checks

All datasets underwent preprocessing for completeness, consistency, and temporal adequacy prior to analysis. That is, observations were checked for presence across the five study countries, country-year values were verified across the four study years, and any missing or inconsistent observations were noted. The GSMA Mobile Connectivity Index (MCI) and ITU household Internet-access measure were strongly correlated in the 10 country-years for which both were available (*r* = 0.945; Table 2), supporting their treatment as convergent measures of digital exposure. Both component measures were nevertheless retained for robustness checks to reduce reliance on a single indicator. The Government AI Readiness Index was validated using the methodology employed in Oxford Insights’ research and in the 2025 edition. Because only 1 year was available for this study, AI readiness was treated as a cross-sectional measure of the institution’s capacity rather than a longitudinal predictor. Such validation procedures allowed for the assessment of measurement validity and the mitigation of the risk of findings being affected by missing observations, inconsistent coverage, and reliance on a single indicator. Convergence of the GSMA and ITU digital exposure measures was also assessed. Among the 10 cases in which both measures were available, the correlation remained high (*r* = 0.95). Therefore, both indicators were considered convergent and indicators of the same construct, rather than distinct predictors, thereby reducing the risk of redundancy. Whenever applicable, the UCDP/PRIO conflict measures and the Afrobarometer measures were used as additional robustness checks. ACLED fatalities were also assessed on a log scale as an additional step to assess whether large country-level differences affect the observed relationships. Hypotheses H1, H2, and H3 were tested according to the direction and strength of the observed relationships, including the positive association between digital information. A positive relationship between manipulation of digital information and security outcomes supports H1; a positive relationship between digital exposure and security outcomes supports H2; and a negative relationship between government readiness for artificial intelligence and security outcomes supports H3. It is important to note that these findings are purely descriptive.

**Table 2:** Bivariate correlations between key predictors and documented security outcomes (ACLED fatalities) strong convergence.

Predictor	*r* (Pearson’s *r*)	*n* (*n*, sample size)	Hypothesis	Direction vs. hypothesis
Digital Information Manipulation Index	0.157	15	H1 (positive)	Consistent (weak)
Digital Information Manipulation Index (log fatalities)	0.232	15	H1 (positive)	Consistent (weak)
Digital Exposure Index	−0.562	15	H2 (positive)	Opposite direction
Government AI Readiness (total score)	−0.419	5	H3 (negative)	Consistent (weak)
Government AI Readiness (Governance pillar only)	−0.069	5	H3 (negative)	Consistent (negligible)
GSMA MCI vs. ITU household access (convergent validity)	0.945	10	n/a	Strong convergence

### 4.8 Limitations of the methods used

Some limitations affect the interpretation of the results. First, the research deliberately uses a small sample size, involving only five countries, and a limited observation period of three to four years, given the variables used. In essence, the correlations identified in the analysis should not be interpreted as evidence of statistical significance or causation, but rather as descriptive indicators of association. Second, the sample selection process is based on population size rather than AI readiness, conflict exposure, or representativeness. The five selected countries were chosen for their ability to provide clear and substantially different examples of large African countries, although this does not mean that the results can be applied to all African countries. Third, the placement of the five cases in the global context should be viewed as contextual benchmarking, not as proof that the sample represents the global situation regarding AI usage. Fourth, the ACLED fatalities include fatalities caused by various conflicts and not just deaths caused by information manipulation through online tools. Fifth, the Government AI Readiness Index is currently only available for 2025.

Sixth, Afrobarometer suffers from limited country coverage, has excluded Egypt and the Democratic Republic of Congo in Round 9, and is therefore used only as an additional source. Seventh, the V-Dem disinformation and hate speech indicators measure information manipulation by governments and political parties, but not AI-enabled disinformation and hate speech. Thus, the former are used as the best quantifiable proxy for persistent information manipulation, not the latter. Eighth, the Government AI Readiness Index fails to capture AI capabilities in the defence sector or private-sector investment and thus does not serve as a comprehensive measure of national AI security capabilities. Moreover, additional aspects such as AI infrastructure, AI skills, AI research, and AI adoption are inconsistently captured across countries and years. Lastly, inconsistencies in data coverage across the seven variables result in an unbalanced design for the analysis. Future research must consider these shortcomings when designing the research framework by using larger samples of countries and time periods.

## 5 Results

This section presents the country-year panel data, organised around the study’s three research objectives. It first describes variation in documented security outcomes, followed by digital exposure, digital information manipulation, and government AI readiness.

### 5.1 Security outcomes: objective one

Conflict fatalities documented in most of the study countries increased during 2023–2025; however, information on fatalities for 2026 is incomplete. The number of fatalities increased most in Nigeria, where the number rose from 8,842 in 2023 to the peak figure of 12,837 in 2025, which represents a 45.2% increase. This increase occurred amid ongoing violence by Boko Haram, ISWAP, and IPOB. Ethiopia had the second-highest increase in fatalities, rising by 65.6% from 3,813 fatalities in 2023 to 6,316 fatalities in 2025. In the Democratic Republic of the Congo (DRC), there was a 31.4% increase in fatalities, from 5,550 in 2023 to 7,290 in 2025, reflecting an escalation of the M23/AFC conflict to “war” according to UCDP. South Africa was the only country among the study countries to register a decrease in fatalities, from 310 in 2023 to 160 in 2025, a 48.4% decrease. Egypt had a significantly lower fatality rate than the four countries; however, the number increased from 31 to 54.

Overall, the data reveal considerable cross-national variation in security outcomes, with Nigeria, Ethiopia, and the DRC experiencing marked increases in documented fatalities, while South Africa recorded a decline and Egypt remained comparatively low. Figure 1 presents the full four-year trend.

**Figure 1:**
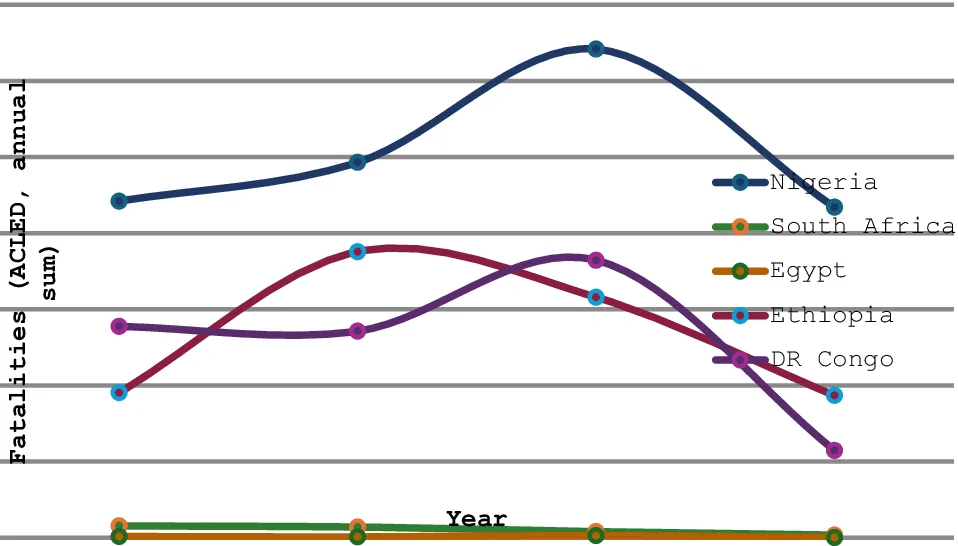
Documented conflict fatalities by country, 2023–2026. Source: Author’s analysis of Armed Conflict Location & Event Data (ACLED), accessed [August, 2026].

### 5.2 Manipulation of digital information and digital exposure

Figure 2 shows significant cross-country differences in the Digital Information Manipulation (DIM) Index over the study period. Ethiopia consistently experienced the highest levels, with the index rising from 1.38 in 2023 to 1.77 in 2024, then declining slightly to 1.62 in 2025. The Democratic Republic of the Congo (DRC) and Egypt maintained intermediate values in the range of 0.6 to 0.9, whereas Nigeria and South Africa exhibited the lowest levels. The index for South Africa remained very close to zero throughout the whole period. Such variations demonstrate significant differences in the extent of information manipulation among governments and political parties across the five countries. However, it should be noted that a relatively low score for South Africa on the DIM does not mean there was no manipulation using AI technology in that country. On the contrary, this difference should be considered in contrast to the high-profile cases of AI-enabled manipulation described in Section 1.1. Publicised isolated incidents may not reflect a persistent trend in information manipulation. Therefore, the DIM Index represents an estimate of such persistent manipulation at the state and party level as opposed to isolated cases of manipulation through AI. It forms the foundation for analysing the relationship between information manipulation and security consequences across countries, as illustrated in Figure 2.

**Figure 2:**
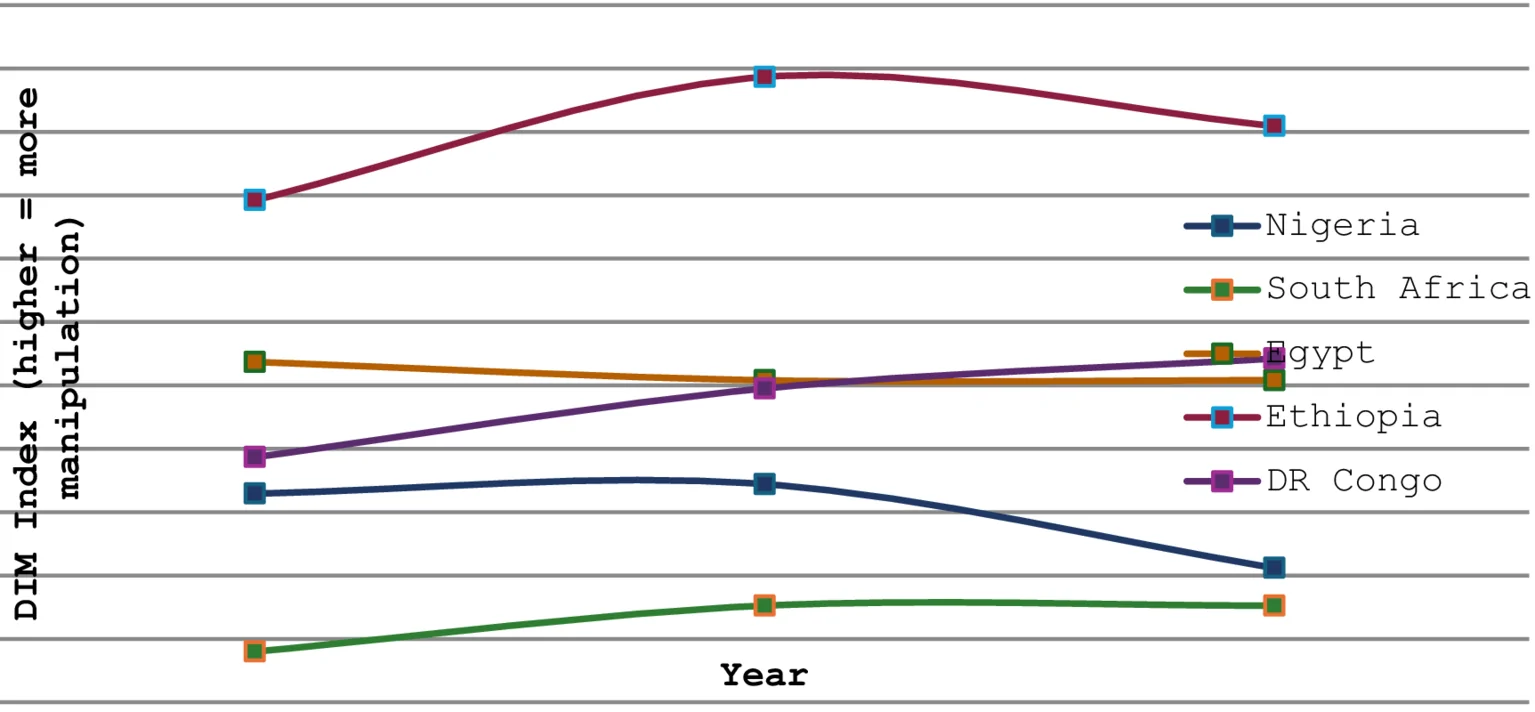
Digital information manipulation index by country, 2023–2025. Source: Author’s analysis of Varieties of Democracy (V-Dem) data, accessed [August, 2026].

Table 3 presents the Digital Exposure Index alongside its two component measures for 2024, the most recent year for which data from both GSMA and ITU are available.

**Table 3:** Digital exposure index by country, 2024.

Country	GSMA MCI	ITU HH internet access (%)	Exposure index
South Africa	71.5	82.1	76.8
Egypt	65.3	75.6	70.4
Nigeria	55.9	44.9	50.4
Ethiopia	42.0	29.2	35.6
DR Congo	28.6	24.2	26.4

The level of digital exposure was highest for South Africa and Egypt in which both surpassed 70 on the composite index, whereas it was much lower for the DRC, being only 26.4. The country rankings were the same for both the GSMA and ITU indices, which is not surprising due to their very high correlation (*r* = 0.95). Such a convergence lends credibility to the use of the composite index as a measure of digital exposure.

### 5.3 Government AI readiness: objective three

Figure 3 shows the scores on the Government AI Readiness Index for 2025. Egypt achieved the highest overall readiness score (59.1), aided by a maximum Policy Capacity score of 100.0 due to its centralised AI strategy at the national level, while the AI Infrastructure score was relatively lower at 47.7. This was followed by South Africa (53.9) and Nigeria (50.8). Again, while Nigeria achieved a very good policy capacity score (80.5), it had a relatively low AI infrastructure score (39.1). Ethiopia (41.6) and the DRC (25.6) had the lowest readiness scores overall. The DRC’s institutional capacity was particularly weak, scoring below 30 on four of six pillars. These results clearly show significant variations across countries in government capacity to develop and govern AI, which forms the foundation for investigating the impact of AI readiness on security outcomes. Figure 3 presents the rankings of the five countries across the six pillars of the Index: Policy Capacity, AI Infrastructure, Governance, Public Sector Adoption, Development and Diffusion, and Resilience. Scores on each pillar range from 0 to 100.

**Figure 3:**
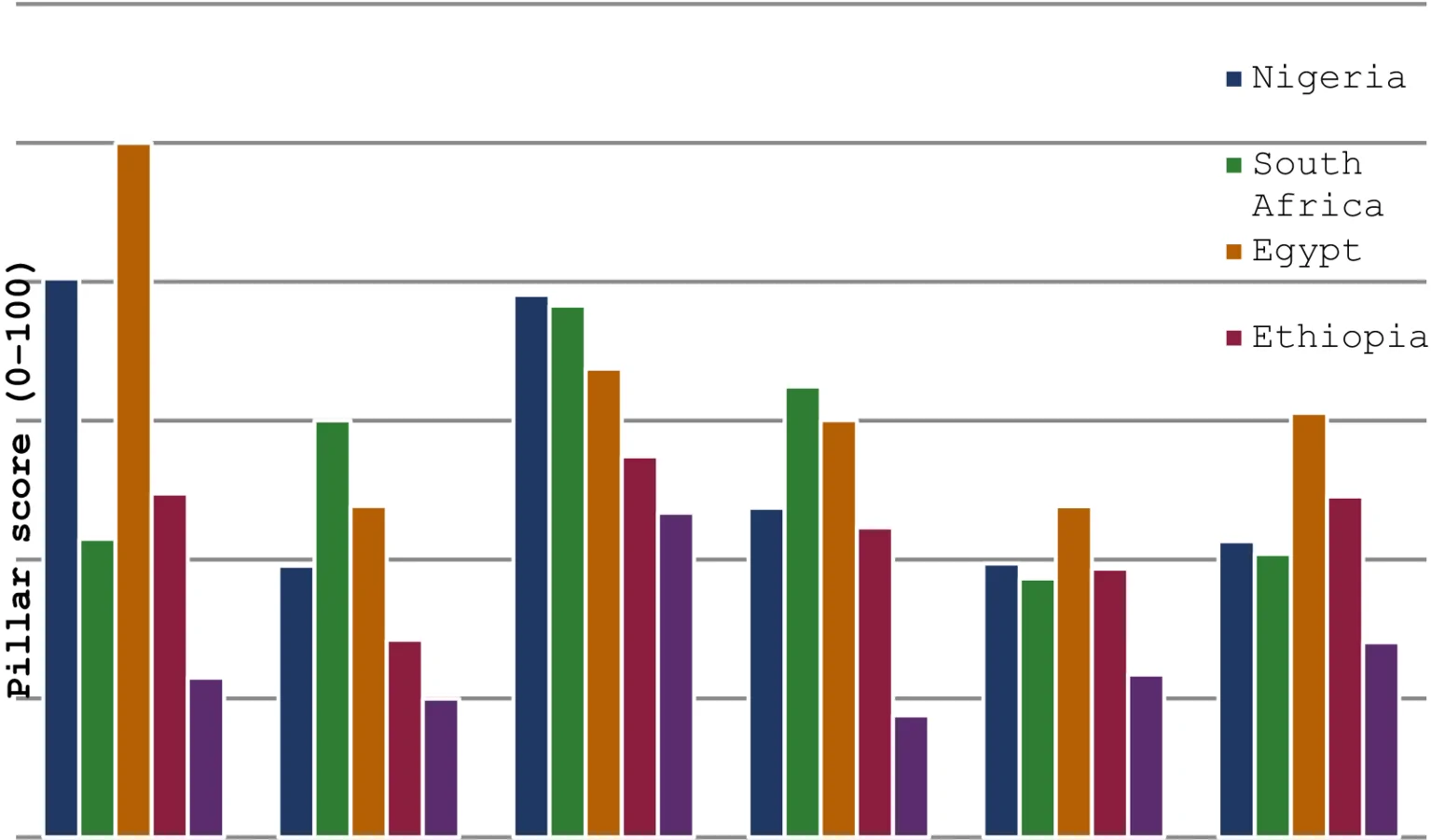
Government AI readiness index pillar scores by country, 2025. Source: Author’s analysis of Oxford Insights’ Government AI Readiness Index 2025 data, accessed [August, 2026].

#### 5.3.1 Positioning the study countries in a global context

The five-country sample is located in the broader international context of AI preparedness. The 2025 Government AI Readiness Index includes assessments of the AI readiness levels of 195 countries across six pillars, providing an international benchmark for analysing the cases studied (Oxford Insights, 2025). Among the countries ranked by the index, Egypt is in 52nd place, South Africa in 65th, Nigeria in 70th, Ethiopia in 91st, and the Democratic Republic of the Congo in 144th. Thus, the five cases under discussion occupy 92 places in the global ranking, ranging from slightly below the top quartile to the bottom of the distribution. Egypt ranks 52nd and therefore falls three places below the top-quartile boundary (49), while the other four cases are even farther from the benchmark. The global comparison shows that the selected countries have very different AI readiness levels on the international scale and cannot be considered as one group. Additionally, none of the five cases is among the best in terms of AI readiness.

### 5.4 Correlation between two variables: hypotheses H1-H3

Table 2 provides an overview of the correlation coefficients among digital manipulation of information, digital exposure, government readiness for Artificial Intelligence, and security outcomes, measured by the number of ACLED fatalities, to test the three hypotheses (H1-H3).

Figure 4 illustrates the relationship between digital exposure and documented security outcomes. The two countries that were least exposed to the Internet, namely, the Democratic Republic of Congo and Ethiopia, also had the greatest number of fatalities due to conflicts during the period under investigation. On the other hand, South Africa and Egypt were characterised by a combination of relatively high levels of digital exposure, together with lower numbers of conflict-related fatalities. The inverse relationship between digital exposure levels and conflict fatalities aligns with the negative correlation between the variables. The relationship, however, has to be treated with some caution. It may simply reflect more complex differences in overall conflict intensity and other related factors, rather than a protective effect of digital exposure per se.

**Figure 4:**
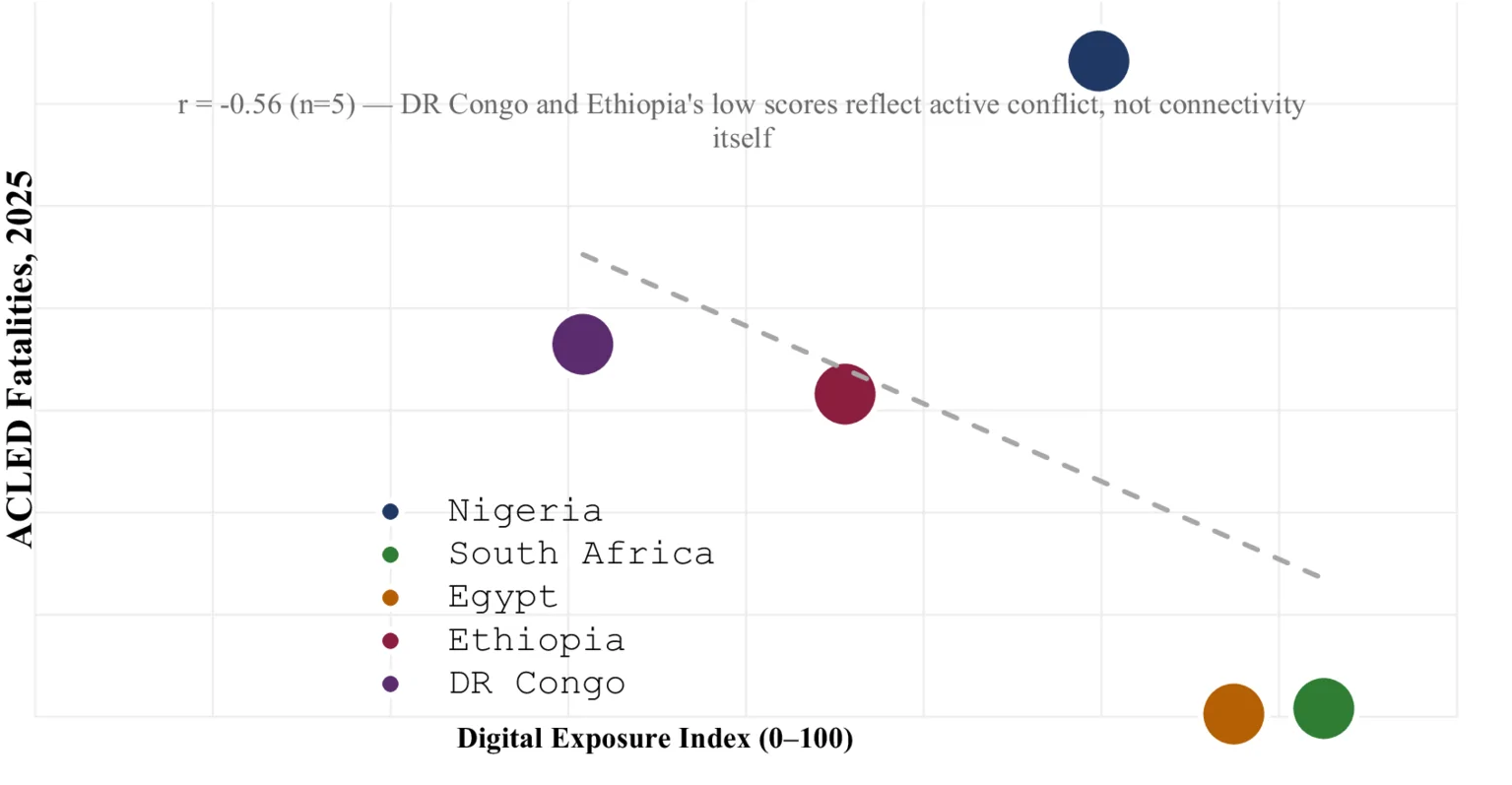
Digital exposure vs. documented security outcomes, 2025. Sources: Author’s analysis of GSMA Mobile Connectivity Index, ITU DataHub, and ACLED data, accessed [August, 2026].

## 6 Discussion

### 6.1 Impact of digital information manipulation on security outcomes

The results lend partial support to Hypothesis 1. While there is a positive correlation between the level of digital information manipulation and conflict fatalities, it is rather weak (*r* = 0.23 for log-fatalities), implying that high levels of digital information manipulation are not necessarily linked to poor security outcomes in the study countries. The case of Ethiopia is illustrative of the predicted trend since the country is characterised by both high levels of digital information manipulation and high conflict fatalities. Nevertheless, Nigeria’s case is striking. Nigeria experienced the highest fatality burden compared to other states under consideration despite the low levels of manipulation described by V-Dem indicators. Thus, it can be assumed that during the study period, the level of insecurity in the studied country was shaped mainly by long-lasting armed conflicts including Boko Haram, ISWAP, and IPOB, rather than by the government and party-related digital information manipulation considered through the lens of V-Dem. In addition, it is important to mention the limitation in measurement as ACLED counts conflict-related deaths regardless of their reasons. Thus, ACLED data cannot determine which specific deaths are linked to information manipulation or insurgencies, nor can it provide evidence of causality.

### 6.2 Digital connectivity and security effects

H2 was rejected in the hypothesised direction. Digital connectivity had a moderate negative relationship with conflict deaths (*r* = −0.56), such that countries with higher connectivity reported lower numbers of deaths. Nevertheless, one cannot assume that digital connectivity per se shields a country from conflict. On the other hand, it appears that this relationship may simply reflect differences in the environments of conflict within the countries considered. For instance, the Democratic Republic of Congo and Ethiopia had low levels of digital connectivity and high levels of intense armed conflict, while South Africa and Egypt had high levels of digital connectivity but low levels of organised violence. As such, it is evident that the levels of conflict, state capacity, and history might be more important factors in security outcomes than connectivity per se.

### 6.3 Government AI readiness and security implications

There is somewhat supportive evidence for H3 in the findings. Government AI readiness was negatively correlated with conflict deaths (*r* = −0.42), consistent with Routine Activity Theory’s proposition that guardianship reduces opportunity. Although the association is modest, the pattern suggests that countries with greater institutional capacity to govern AI may be better positioned to anticipate and manage emerging digital security risks. This finding nevertheless requires considerable caution because AI readiness is observed for only 1 year in the present analysis, and the comparison involves just five countries. Also, the weak negative relationship between conflict deaths and the Governance component (*r* = −0.07) indicates that the overall relationship is not due to regulatory ability alone. It could be due to other facets of state capabilities, including policy coordination, institutional efficiency, and ICTs. This is clearly illustrated by Egypt, which is characterised by the highest levels of AI readiness and policy capabilities despite its low levels of conflict-related deaths.

### 6.4 Cross-cutting observations

The results have uncovered two additional general patterns. The first is that long-standing conflict dynamics appear to be the predominant source of variance in security outcomes across the five selected countries. Nigeria, Ethiopia, and DRC registered the most violent events even with significant variations in the digital manipulation of information, digital exposure, and AI readiness. Therefore, AI-driven manipulation of information seems to occur within the existing political and security dynamics rather than outside them. It is reasonable to assume that the security implications of such manipulation will depend on existing conflict dynamics, institutions, and political tensions. The second general pattern is that digital exposure and government AI readiness are distinct dimensions of states’ digital capacity. In other words, more connectivity does not mean higher institutional preparedness for AI governance. For instance, Egypt registered the highest AI readiness despite lower digital exposure than South Africa, while Nigeria had better policy readiness than South Africa despite lower connectivity.

## 7 Conclusion

This paper examines the links between the manipulation of digital information, digital exposure, government AI preparedness, and the results of security in the five largest countries in Africa using secondary data harmonisation of seven reputable data sources. While the findings confirm some hypotheses, they do not fully support the suggested hypothesis regarding the effect of digital information manipulation on security. Namely, there was a weak positive relationship between digital information manipulation and conflict fatalities. Government AI readiness was negatively associated with conflict fatalities. Moreover, digital exposure also had a negative effect on fatalities; however, the relationship seems to have much to do with differences in conflict contexts rather than the direct role played by connectivity in reducing fatalities. Thus, the results show that persistent armed conflict is a better explanation for cross-national differences in security outcomes than digital information manipulation. Nonetheless, the study shows that digital exposure and government AI readiness are different indicators of national capacities and must not be equated. Regarding the issue raised in section 1.3, this paper provides quantitative cross-country evidence that the effects of digital information manipulation and AI preparedness on security outcomes in Africa are not unambiguous. Digital manipulation and exposure do play a role in determining security outcomes, but their effects are conditioned by conflict situations that cannot be captured by simple bivariate analysis.

## 8 Summary and recommendations

### 8.1 Summary

Based on seven independently verified datasets, the study compiled a comparative panel for Nigeria, South Africa, Egypt, Ethiopia, and the Democratic Republic of the Congo for the period 2023–2026, aligned with the temporal coverage of individual sources. The study explored relationships between digital information manipulation, digital exposure, government AI readiness, and security outcomes. While the findings did not completely confirm the suggested hypotheses, the results indicate a clear pattern. The security implications of digital information manipulation cannot be analysed without accounting for the political, institutional and conflict contexts in which digital technologies operate.

### 8.2 Recommendations

The findings have several implications for policy and practice:

1. AI governance should be integrated into broader national security strategies, particularly in conflict-affected countries such as Ethiopia and the DRC, where relatively limited institutional readiness coincides with persistent insecurity.
2. African Governments should make efforts to strengthen AI governance and move beyond aggregate national rankings and address specific institutional weaknesses, including policy implementation, digital infrastructure, technical expertise, and regulatory oversight.
3. At all government levels, investments should be made in digital literacy and fact-checking initiatives, and early warning mechanisms alongside regulations in order to enhance societal resilience to information manipulation by artificial intelligence.
4. Digital connectivity and AI readiness should be treated as complementary but distinct policy priorities. Expanding internet access can increase digital participation and access to information, but connectivity alone does not guarantee the institutional capacity required to govern emerging AI technologies effectively.
5. Future work will need to expand upon the time series of data available regarding AI readiness and utilise event-level data that helps separate AI manipulation from general conflict. Future studies will also need to focus on other African nations, including larger longitudinal data sets and multivariate panel models.
6. Future research can use fixed- or random-effects panel estimates of H1-H3 once panel data on the Government AI Readiness Index becomes available for several years. The researcher may need to code ACLED/GDELT event narratives to separate instances of manipulation-based unrest from other conflict deaths. The data set can include at least 10 African countries, both those experiencing conflicts and those not. Future work could then formally test the impact of the conflict situation as a possible moderator.

## Data Availability

Publicly available datasets were analyzed in this study. This data can be found here: ACLED Data Download/Export Tool, UCDP Dataset Download Center, V-Dem Dataset, Government AI Readiness Index 2025, GSMA Mobile Connectivity Index, ITU DataHub.
